# Eating patterns of Turkish adolescents: a cross-sectional survey

**DOI:** 10.1186/1475-2891-9-67

**Published:** 2010-12-19

**Authors:** Mehmet Akman, Hülya Akan, Güldal İzbirak, Özlem Tanrıöver, Sırma Mine Tilev, Anıl Yıldız, Simge Tektaş, Ayça Vitrinel, Osman Hayran

**Affiliations:** 1 Department of Family Medicine, Marmara University School of Medicine, Tophanelioğlu Caddesi No:13/15 34662 Altunizade/Istanbul, Turkey; 2 Department of Family Medicine, Yeditepe University Faculty of Medicine, İnönü Mahallesi, Kayışdağı Cad., 26 Ağustos Yerleşimi, 34755 Kadıköy - İstanbul, Turkey; 3 Medical Faculty 3rd yrs students, Yeditepe University Faculty of Medicine, İnönü Mahallesi, Kayışdağı Cad., 26 Ağustos Yerleşimi, 34755 Kadıköy - İstanbul, Turkey; 4 Department of Pediatric Health and Diseases, Yeditepe University Faculty of Medicine, İnönü Mahallesi, Kayışdağı Cad., 26 Ağustos Yerleşimi, 34755 Kadıköy - İstanbul, Turkey; 5 Faculty of Health Sciences, Yeditepe University Faculty of Medicine, İnönü Mahallesi, Kayışdağı Cad., 26 Ağustos Yerleşimi, 34755 Kadıköy - İstanbul, Turkey

## Abstract

**Background:**

Adolescence is a crucial period for development of dietary behaviors that continue into adulthood and influence the risk of chronic diseases later in life. The aim of this study was to determine the eating patterns of adolescents' and their compliance with the Food Guide Pyramid.

**Methods:**

625 students, aged between 11-15 years, from an elementary school in Istanbul, Turkey were enrolled in this cross-sectional survey. A questionnaire of eating patterns (QEP) was administered to all participants. QEP is consisted of questions assessing the knowledge and behaviors on healthy eating, factors affecting food choice, physical activity status and demographical variables. Height and weight of all participants were measured. Physical activity status was determined by questioning about participation in regular sport activities, how much time spent watching TV, playing computer games or doing homework.

**Results:**

The mean age of the participants was 12.15 ± 1.15 and 50.5% were female. According to body mass index (BMI) percentiles, 8.3% (52) were obese and 10.2% were overweight. 51% had breakfast every day and only 1.9% met all the recommendations of the Food Guide Pyramid. Among the participants, 31% have fast food at least once every day and 60.8% skip meals. When participants were asked to rate the factors effecting their food choice according to a 10 point Likert scale, the highest mean scores (high impact on food choice) were for the factors; family, health, body perception, teachers and friends; 7.5 ± 3.1, 7.4 ± 3.1, 6.1 ± 3.2, 4.8 ± 3.3 and 4.2 ± 3.0 respectively. Total mean time spent on all passive activities (TV, computer, reading homework etc) per day was 9.8 ± 4.7 hours.

**Conclusions:**

In this study we have demonstrated that, adolescents do not have healthy eating patterns. Educational interventions should be planned to decrease the health risks attributable to their eating behaviors.

## Background

Adolescence is a crucial period for the development of dietary behaviors; they continue into adulthood and influence the risks of chronic diseases later in life [1-3]. The health of children and adolescents is dependent upon food intake to provide sufficient energy and nutrients to promote optimal physical growth, social and cognitive development [4]. The prevalence of obesity and overweight among 6-19 year old Turkish adolescents has been reported as 3.7-6.8% and 11.5-12.2%, respectively [5,6]. It has been reported that only 1% of the children and adolescents in the United States of America meet all the recommendations of the Food Guide Pyramid (FGP) [3]. Psychosocial and environmental factors play an important role in food choices of adolescents [1,7,8].

The purpose of this study was to determine to which extent adolescents are meeting healthy eating recommendations and to explore associations between background variables, physical activity and healthy eating patterns.

## Methods

This study was designed as a cross-sectional survey. A questionnaire of eating patterns (QEP) was structured by consensus of authors after the review of the relevant literature. QEP administered in an elementary school in Istanbul, Turkey and all students between 11-15 years were enrolled in the study. This public school is sister school of Yeditepe University. Most of the students in this school have poor socioeconomic status. Out of 850 students 795 parents gave written consent for their child to participate. Of these 625 (78.6%) filled in the QEP under supervision of study team. All surveys were administered at the same hour, in the first week of school year. School administration decided the most suitable timing and study team made the necessary preparations accordingly. Study personnel measured height (cm) and weight (kg) of each student. In Turkey all students wear standard suits at school. Weight has been measured with pants on in boys and skirts on in girls and also shirts on for both gender. Shoes and other wears have been taken off. Digital equipment has been used to measure weight. Height has been measured without shoes with a standard stadiometer set on the wall.

According to body mass index (BMI) percentiles [9], the 95th percentile and above was considered as obese and 85th percentile and above was considered as overweight [10-12].

QEP is consisted of knowledge and behavior questions on healthy eating, factors effecting food choice, physical activity status and demographical variables. Each factor that was thought to have an impact on food choice was rated independently according to a 10 point scale (1: least effective to 10: most effective). QEP also explores knowledge and behavior of participants regarding recommendations specified in the Food Guide Pyramid (FGP) [13,14]. Participants were asked to list food groups in the FGP in the order of the recommended consumption frequency and also as their own consumption order taking the previous day as a reference.

The physical activity status was determined by asking about regular participation in sports activities, how much time spent watching TV, playing computer games or doing homework. The National Education Directory of Istanbul Governorship approved the study provided that informed consent is given by both parents and children.

Survey responses were analyzed by SPSS for windows version 11.0. Descriptive statistics were calculated first and then further statistical analyses were conducted to determine the possible associations between participants' demographics, physical activity status and their eating patterns. A chi-square test was used for the comparison of categorical variables and the Student t test or its nonparametric equivalent was used for the comparison of continuous variables.

## Results

The mean age of the participants was 12.15 ± 1.15 and 50.5% (315) were female. Background variables and eating patterns of the participants are shown in Table 1 and 2 (Table 1 and 2). 21.6% of the participants living in the family owned apartment/house. According to BMI percentiles, 8.3% (52) were obese and 10.2% (64) were overweight. 54.1% doing sports regularly. Only 5% of the participants have dinner after 23:00 and 79.8% eat together with their family at the table. 60.8% of the participants had a lesson about healthy eating at school. Eating patterns showed no difference between gender, maternal and paternal education groups. Being obese or overweight and doing sportive activity regularly also have no effect on eating patterns.

**Table 1:** Background variables of the participants

		% (n)
Age groups	11	24.4 (152)
	12	26.9 (168)
	13	20.6 (129)
	14	28.2 (176)

Maternal educational status	Illiterate	4.8 (30)
	Elementary school	39.9 (249)
	University	0.5 (3)

Paternal educational status	Illiterate	0.8 (5)
	Elementary school	41.1(257)
	University	0.6(4)

**Table 2:** Eating patterns of the participants

		% (n)
Main meals per day	1-2	22.0(138)
	3	64.9 (404)
	4-5 or more	12.0(75)

Having breakfast	Every morning	51.0 (319)
	1-2 per week	23.8 (149)
	Never	6.2 (39)

Fast food consumption	Once or more per day	31.5 (197)
	2-6 per week	15.0 (94)
	Once a week/month	34.0 (212)
	Never	16.8 (105)

Choice of snacks	Fruit	47.8(299)
	Cakes and sweets	37.0 (231)
	Vegetables	35.5 (222)
	Fast food	48.6 (304)
	Nuts	18.7(117)

Skipping meal	At least once a day	60.8 (380)

Timing of dinner	Between 17:00-18:59	23.4(146)
	Later than 23:00	0.8(5)

Place of meals	At table with family	79.8(499)
	In front of TV or computer; or while doing homework	13.9(87)

When participants were asked to rate the factors affecting their food choice according to a 10 point scale, the highest mean scores (high impact on food choice) were obtained for the factors; family, health, body perception, teachers and friends; 7.5 ± 3.1, 7.4 ± 3.1, 6.1 ± 3.2, 4.8 ± 3.3 and 4.2 ± 3.0 respectively. There was no statistically significant difference between gender groups regarding factors effecting food choice.

Participants were asked to list the food groups in the FGP in the order of the recommended consumption frequency. Only one (0.2%) participant out of 359, was able to list all the food groups in the correct order. Fats were listed in their correct order by 44.3% of the participants whereas the other correctly listed food groups were 26.4% correct for the vegetables and fruits, 24.3% for the milk, 24% for the meat and 4.6% for the grain groups. When the participants were asked to list the same food groups in the order of own consumption frequencies (n = 369), the percentages in accord with the FGP were; 37.8% consumed meat as recommended serving amounts in the FGP, 27.2% consumed fat as recommended serving amounts in the FGP, 23.5% drank milk as recommended serving amounts in the FGP, 15% ate vegetables and fruits as recommended serving amounts in the FGP and only 11.8% consumed grains as recommended serving amounts in the FGP.

Among the participants, only 1.9% (n = 7) consumed food groups according to servings recommended in the FGP. Of the participants 46.6% listed 1 or none of the food groups in the recommended consumption order, and 35.4% consumed 1 or none of the food groups in the recommended frequency. However, 25.8% of the participants listed 3 or more food groups according to the FGP recommendations but only 23.4% declared that they consume 3 or more food groups as frequently as recommended. Girls seems to know the recommendations regarding vegetable and fruit groups better than boys (60% vs 40%, x^2 ^= 4.41, p = 0.036) and boys seems to know grain group recommendations better than girls (65.5% vs 35.5%; x^2 ^= 4.26, p = 0.039). More girls than boys consume meat group items according to the recommendations (60.4% vs 39.6%; x^2 ^= 10.10, p = 0.001). In our study group, significantly more girls are obese and overweight than boys (11% vs 5.5%, x^2 ^= 6.42, p = 0.011 and 13.3% vs 7.1%, x^2 ^= 6.54, p = 0.011). When participants divided into two groups according to the criteria of listing at least 3 food groups of the FGP in correct order of the recommended consumption frequency, significantly more girls, obese and overweight subjects listed at least 3 food groups correctly compared to boys, non obese and normal weight subjects (Table 3). Between the same groups, there were no significant difference in consuming at least 3 food groups in the FGP according to recommended daily servings.

**Table 3:** Distribution of Food Pyramid Knowledge among study subgroups

	Participants listed at least 3 food groups of the Food Guide Pyramid in the correct recommender order of consumption %(n)	Participants listed 2 or less food groups of the Food Guide Pyramid in the correct recommender order of consumption %(n)	Total %(n)
Male	10.7(33)	89.3(276)	100(309)

Female	16.2(51)	83.8(264)	100(315)

*x^2 ^*= 4.06, p = 0.044			

Obese	28.8(15)	71.2(37)	100(52)

Not obese	12(69)	88(504)	100(573)

*x^2 ^*= 11.57, p = 0.001			

Overweight	25(16)	75(48)	100(64)

Not overweight	12.1(68)	87.9(493)	100(561)

*x^2 ^*= 8.19, p = 0.004			

Among the participants who were doing a sport activity regularly, the mean number of days per week for that sport activity was 1.9 ± 2.0. Mean hours spent daily on the following activities were 2.5 ± 2.3 for watching TV, 1.2 ± 1.2 for watching DVD/video, 1.9 ± 1.8 for computer games, 1.4 ± 1.2 for reading books and 2.3 ± 1.2 for doing homework. Total mean time spent on all these activities was 9.8 ± 4.7.

## Discussion

Our results showed that, the participants had a very low compliance to the FGP (%1.9) and healthy eating patterns were not common. Only 23.4% declared that they consume 3 or more food groups as frequently as recommended. These results were in concordance with the previous literature [3,15].

It has been reported that adolescents do not have regular meals. Fruit and vegetable consumption was below while saturated fat consumption was higher regarding FGP recommendations among adolescents [7,16-18]. In the present study, 27% and 15% of the participants reported that they consume recommended amount of fats and; vegetables and fruits, respectively. Furthermore, meal skipping was very common (61%) and more than one third chose fast food or cakes and sweets as snacks between meals. Nearly one third of the participants consumed fast food once or more daily. Only 51% of the participants stated that they have breakfast every morning. Skipping breakfast was reported to be associated with a high BMI [19-21]. Adolescents who had breakfast every morning represent 56-87% of all adolescents in different European countries [19,20]. The percentage that ate at the table with the family was 80% among the participants. This can be regarded as a positive finding for healthy eating patterns, since family meals have been reported to play an important role in promoting positive dietary intake among adolescents [17,18,22,23]. Associations between the eating behaviors of parents and adolescents, and also between peers and adolescents eating behaviors have been reported [1,17,18,22]. Present study demonstrated that parents had the highest impact on adolescents' food choice, but the peer effect came after the impact of health concerns, body perception and teachers. Highest influence of parents on dietary intake was rather an expected result since majority of our participants ate their meals with their family.

Adolescence is a period in which overall physical activity decreases and activities requiring less physical effort are becoming more dominant. It is recommended for school age children to have more than 60 minutes of physical activity per day [4,7,16,24]. Half of the participants of this study reported that they attend a sports activity regularly. The American Pediatric Academy recommended not exceeding 2 hours per day for TV and computer activities [16,25]. Adolescents in the present study reported spending 3.9 hours per day on average watching TV and on computer activities, which is nearly double of the recommendation.

Another finding of the present study was that females, overweight and obese participants knew the recommendations of the FGP better than their peers, but none of these groups was significantly different in terms of consuming food groups according to recommended servings of FGP. It seems that overweight children and females are more knowledgeable but they do not exhibit better coherence to the recommendations of the FGP. Although data presented did not include any clues to explain why females, obese or overweight participants knew the FGP better, a possible explanation could be that obese, overweight and female subjects were more concerned about their weight and they might be more informed by and/or exposed to advices of their parents and other care givers. We did not ask if they took part in any intervention regarding weight control, but if they did, this intervention would have been coherent with healthy eating principles. On the other hand self perception of the body image might also play a role for the awareness of this group. It has been reported girls are more desirous of a thinner figure than boys [26,27].

Overall low knowledge about the FGP and significant unhealthy eating habits reveal a need of education. Lessons regarding healthy eating should have a place in the curriculum of primary and secondary schools [2,4,7,23]. To create a greater impact, effective educational methods should be applied. To reach this aim, nationwide programs can be organized like healthy canteens, breakfast at school etc. [2,4,7,8].

Enrolling participants from one elementary school is a limitation of this study. Since data of this study were based on participants' declarations, a recall bias may apply.

## Conclusions

In this study we have demonstrated that, adolescents are not having healthy eating patterns and their eating habits do not meet the recommendations of the FGP. Educational interventions should be planned to decrease the health risks attributable to the eating behaviors.

## Competing interests

The authors declare that they have no competing interests.

## Authors' contributions

MA designed the study, performed the statistical analysis and drafted the manuscript. HA, GI and OT participated in the design of the study and helped to draft it. HA, SMT and AY coordinated and collected the data. AV critically evaluated the final version of the manuscript. OH supervised the whole study process, the statistical analysis and evaluated the manuscript for important intellectual content. All authors read and approved the final manuscript.
